# The association between blood albumin level and cardiovascular complications and mortality risk in ICU patients with CKD

**DOI:** 10.1186/s12872-022-02763-x

**Published:** 2022-07-18

**Authors:** Fengming Huang, Jinmao Fan, Xin Wan, Huogen Liu, Yundi Shi, Hailin Shu, Ying Liu, Tongan Lu, Zhenbin Gong, Ling Gu

**Affiliations:** 1 Department of Critical Care Medicine, Mindong Hospital Affiliated to Fujian Medical University, Fuan, 355000 Fujian People’s Republic of China; 2 Cardiovascular Medicine, Mindong Hospital Affiliated to Fujian Medical University, 89 Heshan Road, Fuan, 355000 Fujian People’s Republic of China

**Keywords:** Albumin, Cardiovascular, Mortality, ICU, Chronic kidney disease

## Abstract

**Background:**

Studies involving the association of blood albumin with prognosis in patients with chronic kidney disease (CKD) during intensive care unit (ICU) were scarce.

**Aim:**

We investigated whether reduced blood albumin level independently associated with an increased risk of cardiovascular (CV) complications and 1-year mortality risk in ICU patients with CKD.

**Methods:**

The Medical Information Mart for Intensive Care III (MIMIC-III) database was used. Disease diagnosis and death information among a number of 925 ICU patients with CKD, who have been measured for blood biochemistry, were recorded. Here, multivariable logistic regression Models were structured to evaluate the associations between blood albumin levels (first value on admission, maximum and minimum value during ICU) and risks for CV complications and 1-year mortality among these CKD patients.

**Results:**

In 925 CKD patients, the number of CV complication with heart failure (HF), myocardial infarction (MI) or stroke was 470 (50.8%). 406 (43.9%) patients were dead during the follow-up of 1 year after patients were discharged. Our smooth curve results suggested a curvilinear relation on association between blood albumin level and risk of CV complications. The “inflection point” of blood albumin level that patients were at highest risk of CV complications was 3.4 g/dL. The almost linear relationship with a downward trend was observed on the association between blood albumin level and 1-year mortality risk. We found that reduced blood albumin level contributed to lower risk for CV complications and higher risk for 1-year mortality respectively when blood albumin levels in CKD patients were below 3.4 g/dL. Additionally, albumin therapy had an obvious modifying effect on the independent association, suggesting a possible improved effect of albumin therapy on risk of CV complications and 1-year mortality risk in these CKD patients.

**Conclusions:**

Our study reported that reduced blood albumin levels in CKD patients during ICU were related to lower risk for CV complications and increased risk of 1-year mortality.

**Supplementary Information:**

The online version contains supplementary material available at 10.1186/s12872-022-02763-x.

## Introduction

Chronic kidney disease (CKD) has been considered to be a common health problem, with an estimated prevalence of 13.4% globally [1]. Although in recent years increased medical resources have been allocated for the CKD treatment, life expectancy was still significantly reduced in these CKD patients, compared with individuals with normal kidney function [2]. Importantly, cardiovascular (CV) complications remain the leading cause of death among these CKD patients. A previous meta-analysis of 1,371,990 individuals evaluated the association of nondialysis-dependent CKD with the risk for CV events and all-cause mortality, suggesting a dramatic increase in mortality risk after adjusting other confounding factors [3]. Another meta-analysis including more than 1.4 million subjects demonstrated a significant association of higher albuminuria with risk for cardiovascular disease (CVD) [4]. It can be seen that the CVD risk in CKD patients surpasses the risk of reaching end-stage kidney disease [5, 6]. Thus, it is very significant to find predictors of CV complications or mortality risk in patients with CKD.

As a 69 kDa protein, albumin accounts for > 50% of serum body's composition, which plays an important role in various physiological functions in body [7]. Because blood albumin has exhibited many properties including anti-inflammation, antioxidant, anticoagulant, antiplatelet aggregation and colloidal permeation [8], hypoalbuminemia (HAE) has been regarded as an potentially modifiable risk factor for CVD. Existing epidemiological evidence has suggested that reduced blood albumin levels were related to myocardial infarction (MI), atrial fibrillation (AF), heart failure (HF) and stroke [9–13]. For instance, the Atherosclerosis Risk in Communities (ARIC) study involving 14,506 individuals reported a significant association of reduced blood albumin with the prevalence of ischemic heart disease [9]. Framingham Offspring study demonstrated that serum albumin could be considered as an independent risk factor on predicting first MI in the 4506 individuals with a follow-up for 22 years [10]. The Health ABC Study suggested that reduced serum level of albumin was independently linked to the occurrence of new onset HF with preserved ejection fraction [11]. The Copenhagen City Heart Study suggested that reduced serum levels of albumin were significantly related to AF development in women, independent of other confounding factors [12]. The Northern Manhattan Study involving 2986 individuals suggested that reduced serum level of albumin was an independent risk factor on predicting incident of cardioembolic and cryptogenic stroke [13].

HAE is mainly caused by the imbalance between decreased synthesis of blood albumin in the liver and increased catabolism. Malnutrition and inflammation are considered to be the two main causes of HAE. Previous evidence reported that tumor necrosis factor can mediate the chronic consumption of albumin in patients with malignant tumors, resulting in HAE syndrome [14]. Importantly, inflammation and oxidative stress play important roles in the progression of CV complications in patients with CKD [15–17]. Malnutrition is also common, especially in patients with advanced CKD or ICU inpatients. However, studies involving the association of blood albumin with the prognosis in CKD patients during intensive care unit (ICU) were few. Considering these research backgrounds, we obtained 925 ICU patients with CKD from the Medical Information Mart for Intensive Care III (MIMIC-III) database. We investigated whether reduced blood albumin levels (first value on admission, maximum and minimum value during ICU) independently contributed to increased risks for CV complications and 1-year mortality among these CKD patients.

## Materials and methods

### Study samples

We performed a retrospective study involving ICU patients from MIMIC-III database with a diagnosis of CKD. Our study was consistent with the Strengthening the Reporting of Observational studies in Epidemiology (STROBE) statement [18]. The institutional review boards (IRB) of Beth Israel Deaconess Medical Center (BIDMC) and Massachusetts Institute of Technology (MIT) approved the project and informed consents were exempted due to all patients’ data were anonymized before the data were obtained. All methods were carried out in accordance with relevant guidelines and regulations [18].

**Fig. 1:**
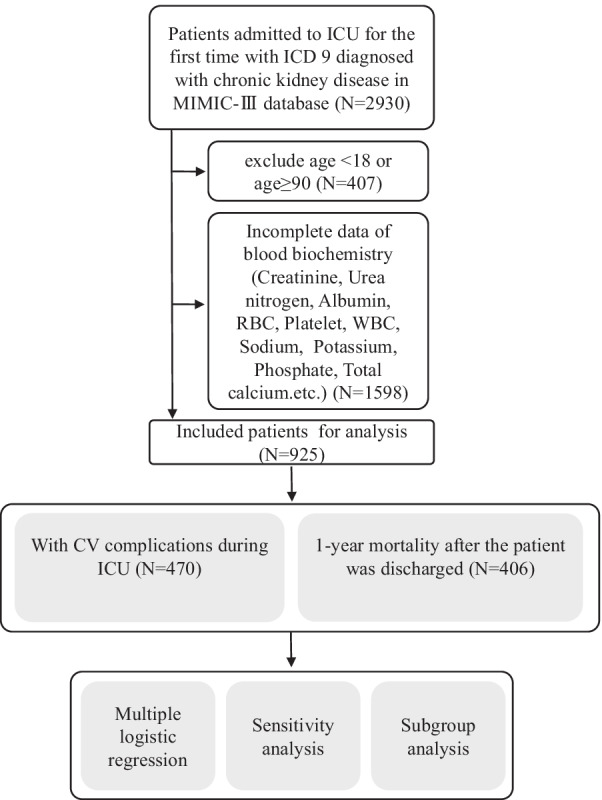
The detailed inclusion for study population

The database that contains clinical information from 46,520 ICU patients from 2002 to 2011, is developed and maintained by the Laboratory for Computational Physiology at MIT [19] and includes clinical data from bedside monitors during ICUs of BIDMC, located in Boston, Massachusetts, USA. ICU nurses verified and recorded the hourly physiological data from the bedside monitors. The database provided demographic characteristics, biochemical examination, intravenous medications and other clinical information. For the purpose of our study, we firstly obtained the data from 2930 patients with CKD. Among these patients, only first ICU admission was included if some individuals had multiple ICU admission records. The disease diagnosis including CKD during ICU was based on the International Classification of Diseases, Ninth Revision (ICD-9) codes that were documented by hospital staffs when patients were discharged. Due to many duplicate record values, the blood biochemical tests including blood albumin and other indicators of the first (1st), maximum (max) and minimum (min) recorded values were used and further analyzed. Finally, these 925 CKD patients who also had no acute kidney injury (AKI) diagnosis during ICU based on ICD-9 codes, were enrolled into our cohort study. The detailed inclusion for these CKD patients is shown in Fig. 1.

### Clinical outcomes

Clinical outcomes of our study were CV complications during ICU treatment and 1-year mortality after included patients were discharged. CV complications were defined as the diagnosis of a composite event including MI, HF or stroke during ICU according to ICD-9. The mortality information was also recorded in the MIMIC-III database. We utilized the death date minus the date of discharge to obtain survival time for each patient.

### Covariates

Demographic characteristics and clinical variables included age, gender, insurance type, ethnicity, blood biochemistry, concomitant diseases and medications during ICU. The insurance in these CKD patients mainly came from government, medicaid, medicare, private and self -pay insurance. Ethnicity mainly contained White, Black, Asian and other. The concomitant diseases mainly included hypertension, diabetes and malignant tumor. The medication during ICU mainly included albumin, β receptor blocker (βRB), statins and heparin.

### Statistical analysis

In all statistical analyses, EmpowerStats 3.0 was used. To infer the association between blood albumin level and clinical outcomes in CKD patients, multivariate regression model was applied to estimate the independent causal effect of blood albumin on CV complications and 1-year mortality by adjusting for the other clinical variables related to the exposure or outcome in our study. Three logistic regression models were established: Model 1 adjusted for age and gender, Model 2 adjusted for age, gender, insurance and ethnicity and Model 3 adjusted for age, gender, insurance, ethnicity, hypertension, diabetes and malignant tumor. Worsening renal function often contributes to a dramatically increased CVD risk and/or short-term death in CKD patients. Therefore, we further used sensitivity analysis to investigated association of blood albumin with risk for CV complications and 1-year mortality by adding “creatinine and uric acid nitrogen” as covariate into the above three models.

Moreover, a single-factor smooth curve analysis was used for evaluating association between blood albumin and CV complications and 1-year mortality. A smoothed curve can fully explore the nonlinear relationship and find the exact “inflection point”. Then a stratification analysis used the “inflection point” of blood albumin level that patients were the highest risk of CV complications or 1-year mortality, as the stratification variable. Then, a conditional logistic regression model was performed to analyzed. *P* value less than 0.05 was statistically significant.

## Results

### Characteristics of CKD patients during ICU

After reviewing admissions in MIMIC-III database, we identified 925 CKD patients admitted to ICU and only patients’ first ICU admissions were analyzed in our study cohort (Fig. 1). The clinical features in the cohort were summarized in Table 1. The number of CV complications including HF, MI and stroke in these CKD patients were 427 (90.85%), 39 (8.30%) and 40 (8.51%), respectively. Patients with CV complications had significantly higher age, prevalence of malignant tumor, rate of used medications (βRB and statins) and 1-year mortality risk, compared with patients without CV complications. Blood biochemistry during ICU were also summarized in Additional file 1.

**Table 1:** Clinical characteristics of ICU patients with CKD (N = 925)

Variables	Without CV complications (N = 455)	With CV complications (N = 470)	*P* value
Age (year)	69.19 (59.20–79.24)	76.55 (67.74–82.83)	< 0.001
Gender (male), n (%)	303 (66.59%)	290 (61.70%)	0.121
*Insurance types*			
Insurance-Government, n (%)	6 (1.32%)	4 (0.85%)	< 0.001
Insurance-Medicaid, n (%)	37 (8.13%)	25 (5.32%)	
Insurance-Medicare, n (%)	297 (65.27%)	369 (78.51%)	
Insurance-Private, n (%)	112 (24.62%)	72 (15.32%)	
Insurance-Self Pay, n (%)	3 (0.66%)	0 (0.00%)	
*Ethnicity*			
Ethnicity-White, n (%)	329 (72.31%)	354 (75.32%)	0.601
Ethnicity-Black, n (%)	55 (12.09%)	50 (10.64%)	
Ethnicity-Asian, n (%)	10 (2.20%)	13 (2.77%)	
Ethnicity-Other, n (%)	61 (13.41%)	53 (11.28%)	
*Concomitant diseases*			
Hypertension, n (%)	36 (7.91%)	35 (7.45%)	0.790
Diabetes, n (%)	27 (5.93%)	42 (8.94%)	0.082
Malignant tumor, n (%)	53 (11.65%)	23 (4.89%)	< 0.001
*CV complications*			
HF, n (%)	–	427 (90.85%)	–
MI, n (%)	–	39 (8.30%)	–
Stroke, n (%)	–	40 (8.51%)	–
*Medication during ICU*			
Albumin, n (%)	98 (21.54%)	85 (18.09%)	0.187
βRB, n (%)	259 (56.92%)	319 (67.87%)	< 0.001
Statins, n (%)	92 (20.22%)	143 (30.43%)	< 0.001
Heparin, n (%)	265 (58.24%)	265 (56.38%)	0.568
Death during ICU, n (%)	88 (19.34%)	109 (23.19%)	0.153
1-year mortality after ICU, n (%)	180 (39.56%)	226 (48.09%)	0.009

-1st: the first laboratory value in ICU; -max: the maximum laboratory value in ICU; -min: the minimum laboratory value in ICU

CV, cardiovascular; ICU, intensive care unit; CKD, chronic kidney disease; HF, heart failure; MI, myocardial infarction; βRB, β Receptor blocker

### Reduced blood albumin level contributed to lower risk for CV complications and higher risk for 1-year mortality

Our logistic regression analysis revealed that reduced albumin (for albumin-1st, OR = 1.885, 95% CI 1.474–2.412, *P* < 0.001; for albumin-max, OR = 1.530, 95% CI 1.201–1.949, *P* < 0.001; for albumin-min, OR = 1.868, 95% CI 1.466–2.379, *P* < 0.001) levels were related to lower risk (Odds Ratio, OR) for CV complications in Model 3 after all covariates including age, gender, insurance, ethnicity, hypertension, diabetes and malignant tumor were controlled (Table 2). Instead, we found that reduced albumin (for albumin-1st, OR = 0.658, 95% CI 0.518–0.835, *P* < 0.001; for albumin-max, OR = 0.786, 95% CI 0.620–0.997, *P* = 0.047; for albumin-min, OR = 0.579, 95% CI 0.456–0.735, *P* < 0.001) levels were associated with an increased risk (OR) of 1-year mortality in Model 3 after all covariates were controlled. Worsening renal function has been confirmed to be an important contributing factor on occurrence of CV complication. Therefore, we further utilized sensitivity analysis by adding “creatinine and uric acid nitrogen” as covariate into the above models. We still observed similar results that lower blood albumin level independently contributed to reduced risk (OR) for CV complications and an increased risk (OR) for 1-year mortality respectively (Table 3).

**Table 2:** Logistic regression analysis of relationship between blood albumin level and risk for CV complications and 1-year mortality risk in ICU patients with CKD

Variables	CV complications		1-year mortality		
	OR (95% CI)	*P* value	OR (95% CI)	*P* value	
*Model 1*					
Albumin-1st	1.882 (1.478, 2.396)	< 0.001	0.650 (0.514, 0.821)	< 0.001	
Albumin-max	1.521 (1.199, 1.928)	< 0.001	0.786 (0.623, 0.993)	0.044	
Albumin-min	1.903 (1.499, 2.415)	< 0.001	0.568 (0.449, 0.718)	< 0.001	
*Model 2*					
Albumin-1st	1.897 (1.487, 2.418)	< 0.001	0.656 (0.517, 0.831)	< 0.001	
Albumin-max	1.526 (1.202, 1.938)	< 0.001	0.789 (0.623, 1.000)	0.049	
Albumin-min	1.913 (1.505, 2.430)	< 0.001	0.571 (0.451, 0.724)	< 0.001	
*Model 3*					
Albumin-1st	1.885 (1.474, 2.412)	< 0.001	0.658 (0.518, 0.835)	< 0.001	
Albumin-max	1.530 (1.201, 1.949)	< 0.001	0.786 (0.620, 0.997)	0.047	
Albumin-min	1.868 (1.466, 2.379)	< 0.001	0.579 (0.456, 0.735)	< 0.001	

Model 1: Adjusted for age and gender

Model 2: Adjusted for age, gender, insurance and ethnicity

Model 3: Adjusted for age, gender, insurance, ethnicity, hypertension, diabetes and malignant tumor

-1st: the first laboratory value in ICU; -max: the maximum laboratory value in ICU; -min: the minimum laboratory value in ICU

CV, cardiovascular; ICU, intensive care unit; CKD, chronic kidney disease

**Table 3:** Sensitivity analysis of relationship between blood albumin level and risk for CV complications and 1-year mortality risk in ICU patients with CKD

Variables	CV complications		1-year mortality	
	OR (95% CI)	*P* value	OR (95% CI)	*P* value
*Model 1*				
Albumin-1st	1.878 (1.473, 2.393)	< 0.001	0.650 (0.512, 0.824)	< 0.001
Albumin-max	1.496 (1.179, 1.899)	< 0.001	0.742 (0.583, 0.944)	0.015
Albumin-min	1.909 (1.499, 2.432)	< 0.001	0.484 (0.377, 0.621)	< 0.001
*Model 2*				
Albumin-1st	1.894 (1.484, 2.417)	< 0.001	0.659 (0.518, 0.838)	< 0.001
Albumin-max	1.503 (1.183, 1.910)	< 0.001	0.749 (0.587, 0.956)	0.020
Albumin-min	1.916 (1.502, 2.443)	< 0.001	0.489 (0.380, 0.628)	< 0.001
*Model 3*				
Albumin-1st	1.882 (1.470, 2.410)	< 0.001	0.661 (0.519, 0.843)	< 0.001
Albumin-max	1.508 (1.182, 1.923)	< 0.001	0.748 (0.585, 0.956)	0.020
Albumin-min	1.871 (1.463, 2.392)	< 0.001	0.497 (0.386, 0.640)	< 0.001

Model 1: Adjusted for age, gender, creatinine and urea nitrogen

Model 2: Adjusted for age, gender, insurance, ethnicity, creatinine and urea nitrogen

Model 3: Adjusted for age, gender, insurance, ethnicity, hypertension, diabetes, malignant tumor, creatinine and urea nitrogen

-1st: the first laboratory value in ICU; -max: the maximum laboratory value in ICU; -min: the minimum laboratory value in ICU

CV, cardiovascular; ICU, intensive care unit; CKD, chronic kidney disease

### Curvilinear relation analysis of blood albumin with risk of CV complications and 1-year mortality risk

**Fig. 2:**
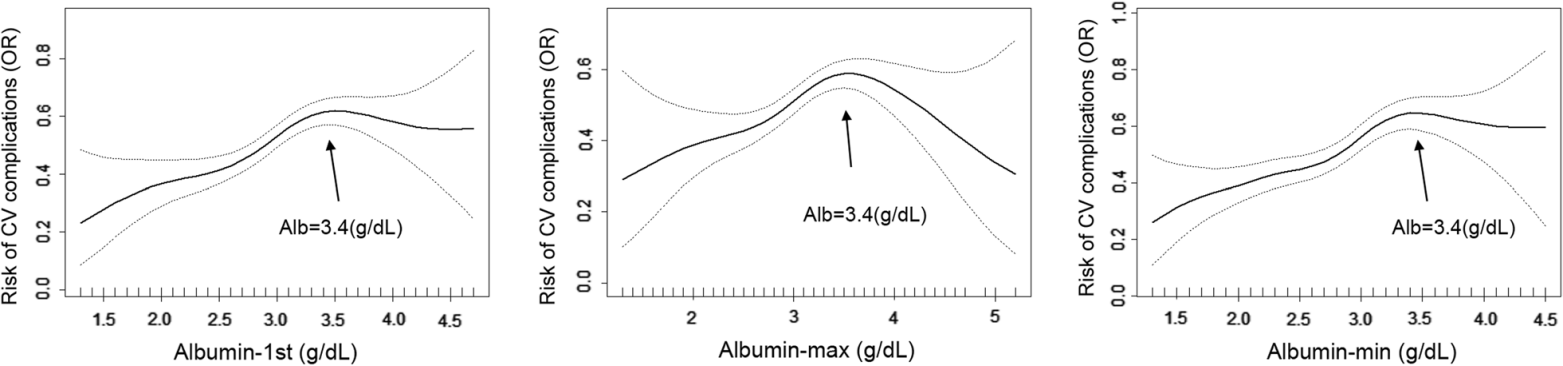
Single-factor smooth curve on association between blood albumin level and risk of CV complications in CKD patients

**Fig. 3:**
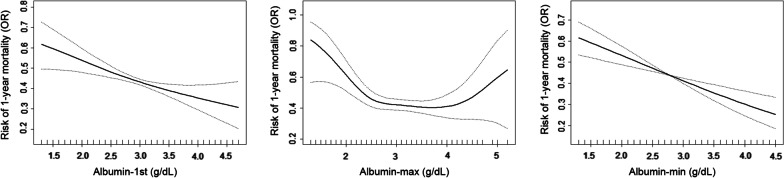
Single-factor smooth curve on association between blood albumin level and risk of 1-year mortality in CKD patients

Single-factor smooth curve was structured by using blood albumin level as X axis and using both risks of CV complications and 1-year mortality Y axis respectively. Our results suggested a curvilinear relation on association between serum albumin levels and risk of CV complications (Fig. 2). We found the “inflection point” of blood albumin level that patients were at highest risk of CV complications was 3.4 g/dL. The almost linear relationship with a downward trend was observed on the association between blood albumin level and 1-year mortality risk, seen in Fig. 3.

Based to the “inflection point” (blood albumin level = 3.4 g/dL), we further divided all CKD patients into two groups by using albumin as categorical variable (patients with blood albumin level < 3.4 g/dL and with blood albumin level ≥ 3.4 g/dL). Our results suggested that lower albumin (for albumin-1st, OR = 2.091, 95% CI 1.420–3.079, *P* < 0.001; for albumin-max, OR = 1.801, 95% CI 1.155–2.809, *P* = 0.009; for albumin-min, OR = 1.985, 95% CI 1.417–2.780, *P* < 0.001) levels still contributed to a reduced risk (OR) for CV complications after all covariates were adjusted in patients with blood albumin level < 3.4 g/dL (Table 4). Similarly, we still observed that lower albumin (for albumin-1st, OR = 0.610, 95% CI 0.419–0.889, *P* = 0.010; for albumin-max, OR = 0.508, 95% CI 0.324–0.796, *P* = 0.003; for albumin-min, OR = 0.566, 95% CI 0.406–0.788, *P* < 0.001) levels contributed to with an increased risk (OR) of 1-year mortality after these covariates were adjusted in patients with blood albumin level < 3.4 g/dL. However, there were not significant associations between blood albumin levels with risk (OR) of CV complications and 1-year mortality risk (OR) in patients with blood albumin level ≥ 3.4 g/dL.

**Table 4:** Relationship between blood albumin level and risk for CV complications and 1-year mortality risk in ICU patients with CKD by using albumin (= 3.4 g/dL) as categorical variable

Variables		CV complications			1-year mortality		
		OR (95% CI)	*P* value	P*	OR (95% CI)	*P* value	P*
Albumin-1st	< 3.4 g/dL	2.091 (1.420, 3.079)	< 0.001	0.003	0.610 (0.419, 0.889)	0.010	0.491
	≥ 3.4 g/dL	0.364 (0.123, 1.077)	0.068		0.904 (0.320, 2.551)	0.849	
Albumin-max	< 3.4 g/dL	1.801 (1.155, 2.809)	0.009	0.001	0.508 (0.324, 0.796)	0.003	0.021
	≥ 3.4 g/dL	0.411 (0.185, 0.913)	0.029		1.288 (0.623, 2.664)	0.494	
Albumin-min	< 3.4 g/dL	1.985 (1.417, 2.780)	< 0.001	0.017	0.566 (0.406, 0.788)	< 0.001	0.833
	≥ 3.4 g/dL	0.274 (0.059, 1.276)	0.099		0.783 (0.150, 4.082)	0.772	

Adjusted for age, gender, insurance, ethnicity, hypertension, diabetes and malignant tumor

P* for interaction in two subgroup

CV, cardiovascular; ICU, intensive care unit; CKD, chronic kidney disease

### Medication-related subgroup analysis for association between blood albumin level with risk of CV complications and 1-year mortality risk

Albumin therapy had an obvious modifying effect (for albumin-max, *P* value for interaction = 0.025; for albumin-min, *P* value for interaction = 0.025) on the independent association blood albumin level with risk of CV complications. Subgroup analysis suggested that lower albumin (for albumin-max, OR = 0.957, 95% CI 0.582–1.572, *P* = 0.861; for albumin-min, OR = 1.140, 95% CI 0.647–2.007, *P* = 0.650) levels did not contribute to reduced risk (OR) for CV complications in patients with albumin therapy (Table 5). Consistently, albumin therapy also had an obvious modifying effect (for albumin-1 st, *P* value for interaction = 0.009; for albumin-max, *P* value for interaction = 0.006; for albumin-min, *P* value for interaction = 0.032) on the independent association between blood albumin and 1-year mortality risk (OR). We found that reduced albumin (for albumin-1st, OR = 1.143, 95% CI 0.678–1.928, *P* = 0.615; for albumin-max, OR = 1.289, 95% CI 0.805–2.065, *P* = 0.290; for albumin-min, OR = 0.859, 95% CI 0.499–1.481, *P* = 0.585) levels did not contribute to reduced 1-year mortality risk (OR) in patients with albumin therapy (Table 5). However, in patients without albumin therapy, reduced blood albumin level still contributed to lower risk (OR) for CV complications and increased risk (OR) for 1-year mortality. These results suggested that albumin therapy might lower risk of CV complications and lower 1-year mortality risk in ICU patients with CKD. Additionally, therapy including used βRB, statins, heparin had obvious modifying effect on the independent associations, suggesting that these drugs did not improve risk of CV complications and 1-year mortality risk in these CKD patients.

**Table 5:** Medication-related subgroup analysis for association between blood albumin level with risk of CV complications and 1-year mortality risk

Variables	Medications	CV complications			1-year mortality		
		OR (95% CI)	*P* value	P*	OR (95% CI)	*P* value	P*
Albumin-1st	Albumin	1.242 (0.718, 2.150)	0.439	0.064	1.143 (0.678, 1.928)	0.615	0.009
	NO Albumin	2.131 (1.605, 2.829)	< 0.001		0.557 (0.422, 0.734)	< 0.001	
	βRB	2.173 (1.577, 2.994)	< 0.001	0.142	0.737 (0.545, 0.997)	0.048	0.193
	NO βRB	1.576 (1.055, 2.354)	0.026		0.520 (0.343, 0.788)	0.002	
	Statins	2.417 (1.439, 4.059)	< 0.001	0.250	0.573 (0.341, 0.966)	0.036	0.500
	NO statins	1.675 (1.256, 2.233)	< 0.001		0.706 (0.536, 0.930)	0.013	
	Heparin	1.670 (1.213, 2.301)	0.002	0.239	0.732 (0.533, 1.004)	0.053	0.286
	NO Heparin	2.294 (1.538, 3.421)	< 0.001		0.578 (0.398, 0.840)	0.004	
Albumin-max	Albumin	0.957 (0.582, 1.572)	0.861	0.025	1.289 (0.805, 2.065)	0.290	0.006
	NO Albumin	1.847 (1.383, 2.467)	< 0.001		0.628 (0.473, 0.834)	0.001	
	βRB	1.687 (1.228, 2.316)	0.001	0.271	0.852 (0.627, 1.157)	0.304	0.418
	NO βRB	1.393 (0.937, 2.070)	0.102		0.687 (0.462, 1.022)	0.064	
	Statins	1.970 (1.171, 3.313)	0.011	0.173	0.547 (0.318, 0.943)	0.030	0.123
	NO statins	1.383 (1.043, 1.835)	0.025		0.877 (0.668, 1.150)	0.341	
	Heparin	1.393 (1.023, 1.896)	0.035	0.447	0.825 (0.607, 1.122)	0.220	0.73
	NO Heparin	1.782 (1.188, 2.671)	0.005		0.768 (0.522, 1.130)	0.181	
Albumin-min	Albumin	1.140 (0.647, 2.007)	0.650	0.025	0.859 (0.499, 1.481)	0.585	0.032
	NO Albumin	2.139 (1.619, 2.825)	< 0.001		0.508 (0.385, 0.669)	< 0.001	
	βRB	2.157 (1.570, 2.962)	< 0.001	0.162	0.651 (0.480, 0.884)	0.006	0.156
	NO βRB	1.607 (1.083, 2.386)	0.019		0.458 (0.304, 0.691)	< 0.001	
	Statins	2.165 (1.327, 3.531)	0.002	0.510	0.419 (0.247, 0.712)	0.001	0.197
	NO statins	1.704 (1.277, 2.273)	< 0.001		0.655 (0.497, 0.864)	0.003	
	Heparin	1.737 (1.247, 2.421)	0.001	0.435	0.563 (0.402, 0.788)	< 0.001	0.991
	NO Heparin	2.085 (1.440, 3.018)	< 0.001		0.592 (0.418, 0.840)	0.003	

Adjusted for age, gender, insurance, ethnicity, hypertension, diabetes and malignant tumor

P* for interaction in two subgroup

CV: cardiovascular; ICU: intensive care unit; CKD: chronic kidney disease

## Discussion

CKD patients tend to be accompanied with higher prevalence of traditional risk factors (aging, smoking, insulin resistance/diabetes, hypertension and dyslipidemia), which is particularly important to lead to atherosclerotic diseases in early stages of CKD [20, 21]. These risk factors not only contribute to the development of cardiovascular and cerebrovascular diseases, but also promote CKD progression due to their effect on kidney artery stenosis and nephrosclerosis [22]. However, high risk of CV complications in CKD can only be partially explained these traditional risk factors, as shown by data from the Cardiovascular Health Study (CHS) trials and ARIC study [23, 24]. The other specific aspects (nontraditional risk factors) in CKD have not fully been solved. For example, oxidative stress-inflammatory reaction is an important process in the progress of CKD, which has been considered to be a chronic inflammatory disease caused by various etiologies [24, 25]. Existing evidence showed that proinflammatory factors in circulating blood progressively increased with decreased renal function [26]. In our cohort study, our results showed that reduced blood levels of albumin were related to an increased risk of 1-year mortality in CKD patients, after adjustment for important identified confounders, which is consistent with the previous results that HEA sharply contributed to increased risk of all-cause mortality in other various populations. However, reduced serum levels of albumin were also associated with a lower risk for CV complications (MI, HF and stroke) during ICU among these patients.

Serum albumin has many physiological properties [27], which can carry lots of exogenous and endogenous substances including hormones, vitamins, fatty acids, inorganic ions bilirubin, drugs and other molecules. Importantly, albumin in circulating blood has been regarded as the most important antioxidant [9, 28]. Albumin contains more than 80% of total thiols in circulating blood for scavenging reactive oxygen and nitrogen species and has significantly anti-inflammatory effects on body [27, 29]. Serum albumin can also carry some substances such as nitric oxide and bilirubin, which indirectly provides additional protection against oxidative stress. Indeed, because of these rotective properties of blood albumin, HAE has been regarded as a potentially independent risk factor for CVD. For instance, in a previous prospective study suggested that HAE can independently contributed to higher risk for cardiovascular (*p* = 0.037) and overall (*p* = 0.048) mortality in 734 patients with stable coronary artery disease [30]. It has been also established that HAE is an independently prognostic factor in HF patients, independent of other prognostic factors, such as B-type natriuretic peptides, renal failure and anemia [30–35]. Bonilla-Palomas et al. found that HAE was related to raised risk of in-hospital death (*p* = 0.04) and long-term mortality (*p* = 0.02), after adjusting for B-type natriuretic peptides and liver function in a observational study involving 362 patients who were hospitalized for acute systolic and diastolic HF [35]. Furthermore, there were evidence supported that HAE can be act as a strong prognostic factor for stroke in many observational studies [36–38], which supported the prognostic value of blood albumin for detecting stroke risk, irrespective of other specific or well-established risk factors. Also, HAE has been reported having a good prognostic value for predicting risks of AF, essential and primary pulmonary hypertension. Hemoglobin and serum albumin tend to decrease in patients with malignant diseases simultaneously, reduced hemoglobin levels have been demonstrated to be useful to predict the prognosis and inform the decision-making in patients with acute cardiovascular diseases [39]. In our study, our results suggested that reduced blood albumin levels (less than 3.4 g/dL) were associated with higher risk of 1-year mortality among ICU patients with CKD, while the significant association disappeared in CKD patients with albumin levels ≥ 3.4 g/dL, which is consistent with previous conclusions that HAE can be regarded as good prognostic factor for predicting risk of long-term all-cause mortality [30–38]. However, we were surprised to observe that reduced albumin levels contributed to lower risk of CV complications, which is inconsistent with previous studies. This difference may be due to the fact that our study samples were obtained from ICU patients, whose life condition were poor that it affects our short-term results. Another reasonable reason is that only single CV event with one specific disease was included as the endpoint in previous studies, which is not consistent our results that a composite event including MI, AF, HF and stroke was used for analysis. Additionally, the main general population (relatively healthy participants) were included on previous studies for analyzing the relationship between albumin and prognosis, but there were few studies conducted on CKD population. Further research may be needed to verify our explanation in future.

Our results also suggested that albumin therapy had an obvious modifying effect on the independent association between blood albumin and 1-year mortality risk. Increased albumin levels did contribute to reduced 1-year mortality risk in patients without albumin therapy but not in those with albumin therapy. The beneficial effect of increased blood albumin in coronary artery disease, HF, AF, stroke and other CVD may primarily refer to its antioxidant, anti-inflammatory, antiplatelet aggregation and electrophysiological activation [40]. Generally known, serum albumin is the most abundant antioxidant in peripheral blood, and oxidative stress-inflammatory response has emerged as an important pathways linking to pathological process of various CVD [41]. Furthermore, there was study reported that oxidative stress-inflammatory response may be related to pathological development of venous thromboembolism [42]. HEA may also contribute to the worsening myocardial edema that has been observed in ischemic heart disease and HF [42–44].

In this retrospective study, we firstly included ICU individual with CKD from MIMIC-III database and reported that reduced blood albumin concentration independently contributed to higher risk of 1-year mortality and lower risk for CV complications during ICU treatment, which further expanded CKD-related studies on prediction of blood albumin for CV events and mortality risk. Interestingly, these significant associations did disappear in patients with serum levels albumin more than 3.4 g/dL. Importantly, we further observed that albumin therapy could reduce long-term mortality risk in these CKD patients. However, some limitations also need to be noted. Firstly, although renal function (blood creatinine and urea nitrogen) have been corrected in our sensitivity analysis, we did not provided unmeasured confounders such as specific clinical classification (CKD1-5) and dialysis history that were critical variables linking to CV complications and prognosis in CKD patients. Secondly, CV complications were diagnosed during ICU hospitalization in our study, while the history of CVD before admission is uncertain and may cause to outcome bias, which might explain for positive association that reduced blood albumin contributed to lower risk of CV complications. Thirdly, the time interval between the measurement of blood albumin and the occurrence of our clinical outcomes was not clear, so we included the first, maximum and minimum values of blood albumin after entering the ICU for analysis. Fourthly, we have not included the variable “AKI”, which is extremely frequent in ICU patients who needed ICU care. The impact of AKI on this study is necessary to confirmed in the future. Fifthly, despite from different races, these ICU patients also led to a poor extrapolation of our research findings.

## Conclusion

Our results firstly reported significant relationships between blood albumin levels and risk of CV complications and 1-year mortality risk, independent of renal function, malignancy and other confounding factors in ICU patients with CKD. Interestingly, these significant associations did disappear when serum levels albumin were more than 3.4 g/dL. The reducing blood albumin in HEA patients (blood albumin < 3.5 g/dL) might be a useful biomarker on predicting poor prognosis in CKD population, probably more reflecting activation of oxidative stress-multiple inflammatory mediators.

## Supplementary Information

**Additional file 1**. Biochemical parameters of ICU patients with CKD (N = 925).

## Data Availability

We performed a retrospective study involving ICU patients from MIMIC-III database with a diagnosis of CKD. The datasets generated during the current study are available in database (https://mimic.mit.edu/).
