# Supplementary material for: The association between blood albumin level and cardiovascular complications and mortality risk in ICU patients with CKD
Source: BMC Cardiovasc Disord. 2022 Jul 18;22:322. doi: 10.1186/s12872-022-02763-x (PMC9295487; doi:10.1186/s12872-022-02763-x)
Supplement: Supplementary file 1 — Additional file 1. Biochemical parameters of ICU patients with CKD (N = 925). [file 12872_2022_2763_MOESM1_ESM.doc]

| **Additional file 1.** Biochemical parameters of ICU patients with CKD (N=925) | | | | |
| --- | --- | --- | --- | --- |
| **Variables** | | **Without CV complications (N=455)** | **With CV complications (N=470)** | ***P* value** |
| Creatinine (mg/dL) | 1st | 1.90 (1.40-3.10) | 1.80 (1.40-2.60) | 0.078 |
|  | max | 2.60 (1.80-4.15) | 2.40 (1.80-3.68) | 0.246 |
|  | min | 1.30 (1.00-1.90) | 1.30 (1.00-1.80) | 0.591 |
| Urea nitrogen (mg/dL) | 1st | 38.00 (24.00-59.00) | 40.50 (27.00-60.00) | 0.094 |
|  | max | 58.00 (37.00-86.50) | 61.00 (42.00-86.75) | 0.177 |
|  | min | 23.00 (14.00-35.00) | 28.00 (18.00-38.75) | <0.001 |
| Albumin (g/dL) | 1st | 2.80 (2.50-3.30) | 3.10 (2.70-3.48) | <0.001 |
|  | max | 3.00 (2.70-3.45) | 3.20 (2.80-3.50) | <0.001 |
|  | min | 2.70 (2.20-3.00) | 2.90 (2.50-3.30) | <0.001 |
| RBC (m/uL) | 1st | 3.29 (2.88-3.65) | 3.38 (2.98-3.89) | 0.004 |
|  | max | 3.63 (3.33-4.02) | 3.75 (3.44-4.17) | <0.001 |
|  | min | 2.69 (2.36-3.03) | 2.77 (2.53-3.17) | <0.001 |
| Platelet (K/uL) | 1st | 182.00 (116.00-262.50) | 189.50 (142.25-259.00) | 0.099 |
|  | max | 295.00 (199.50-411.50) | 280.00 (198.75-381.00) | 0.479 |
|  | min | 131.00 (77.00-194.00) | 135.50 (97.00-187.25) | 0.223 |
| WBC (K/uL) | 1st | 11.20 (7.60-16.10) | 10.80 (7.70-15.28) | 0.727 |
|  | max | 15.70 (11.55-21.40) | 15.65 (11.13-21.27) | 0.846 |
|  | min | 6.80 (4.80-8.80) | 6.85 (5.40-9.08) | 0.117 |
| PTT (sec) | 1st | 33.00 (28.25-41.40) | 34.00 (28.50-44.80) | 0.141 |
|  | max | 41.00 (32.10-74.80) | 48.50 (35.00-110.40) | <0.001 |
|  | min | 28.20 (25.20-31.80) | 28.00 (25.20-31.40) | 0.789 |
| PT (sec) | 1st | 14.80 (13.40-17.25) | 15.50 (13.60-18.70) | 0.009 |
|  | max | 16.50 (14.30-23.65) | 18.30 (15.10-26.27) | <0.001 |
|  | min | 13.10 (12.30-14.50) | 13.45 (12.33-14.80) | 0.092 |
| INR-PT | 1st | 1.30 (1.20-1.60) | 1.40 (1.20-1.70) | 0.009 |
|  | max | 1.50 (1.30-2.30) | 1.70 (1.30-2.60) | 0.002 |
|  | min | 1.10 (1.10-1.30) | 1.20 (1.10-1.30) | 0.107 |
| Glucose (mg/dL) | 1st | 130.00 (104.00-172.50) | 136.00 (108.00-183.00) | 0.078 |
|  | max | 200.00 (154.00-270.00) | 204.00 (162.25-270.00) | 0.375 |
|  | min | 84.00 (70.00-97.00) | 83.50 (68.00-96.00) | 0.417 |
| Sodium (mEq/L) | 1st | 138.00 (135.00-142.00) | 138.00 (135.00-141.00) | 0.330 |
|  | max | 144.00 (141.00-148.00) | 144.00 (141.00-148.00) | 0.373 |
|  | min | 134.00 (131.00-137.00) | 134.00 (131.00-138.00) | 0.206 |
| Chloride (mEq/L) | 1st | 107.00 (102.00-111.00) | 105.00 (101.00-109.00) | <0.001 |
|  | max | 113.00 (108.00-117.00) | 110.00 (107.00-115.00) | <0.001 |
|  | min | 100.00 (97.00-104.00) | 99.00 (94.00-103.00) | <0.001 |
| Potassium (mEq/L) | 1st | 4.40 (3.90-5.10) | 4.30 (3.80-4.90) | 0.129 |
|  | max | 5.10 (4.60-5.70) | 5.10 (4.60-5.70) | 0.829 |
|  | min | 3.40 (3.10-3.70) | 3.40 (3.10-3.70) | 0.817 |
| Magnesium (mg/dL) | 1st | 1.90 (1.70-2.30) | 2.10 (1.80-2.40) | <0.001 |
|  | max | 2.50 (2.30-2.80) | 2.60 (2.30-2.80) | 0.015 |
|  | min | 1.70 (1.50-1.90) | 1.80 (1.60-2.00) | <0.001 |
| Phosphate (mg/dL) | 1st | 3.90 (3.10-5.00) | 4.00 (3.30-5.00) | 0.451 |
|  | max | 5.10 (4.15-6.40) | 5.10 (4.20-6.30) | 0.578 |
|  | min | 2.40 (1.85-3.10) | 2.50 (2.00-3.20) | 0.054 |
| Total calcium (mg/dL) | 1st | 8.10 (7.60-8.60) | 8.30 (7.80-8.80) | <0.001 |
|  | max | 8.90 (8.50-9.40) | 9.00 (8.60-9.47) | 0.177 |
|  | min | 7.50 (7.00-8.00) | 7.75 (7.30-8.20) | <0.001 |
| PO_2_ (mmHg) | 1st | 109.00 (73.00-218.00) | 108.50 (73.00-235.25) | 0.843 |
|  | max | 188.00 (112.00-297.50) | 208.00 (122.25-352.00) | 0.013 |
|  | min | 65.00 (43.00-90.00) | 61.00 (40.00-80.00) | 0.005 |
| PCO_2_ (mmHg) | 1st | 38.00 (32.00-44.00) | 40.00 (34.00-48.00) | <0.001 |
|  | max | 46.00 (38.00-54.50) | 48.00 (41.00-59.00) | <0.001 |
|  | min | 31.00 (27.00-36.50) | 32.00 (28.00-38.00) | 0.050 |
| Bicarbonate (mEq/L) | 1st | 21.00 (18.00-24.00) | 23.00 (20.00-26.00) | <0.001 |
|  | max | 28.00 (24.00-31.00) | 30.00 (27.00-33.00) | <0.001 |
|  | min | 18.00 (15.00-21.00) | 20.00 (17.00-23.00) | <0.001 |
| 1st: the First laboratory value in ICU; max: the Maximum laboratory value in ICU; min: the Minimum laboratory value in ICU.  CV: cardiovascular; ICU: intensive care unit; CKD: chronic kidney disease; RBC: red blood cell; WBC: white blood cell; PTT: partial thromboplastin time; PT: prothrombin time; INR-PT: international normalized ratio of prothrombin time. | | | | |
